# Therapeutic Potential of Heme Oxygenase-1/Carbon Monoxide in Lung Disease

**DOI:** 10.1155/2012/859235

**Published:** 2012-02-01

**Authors:** Myrna Constantin, Alexander J. S. Choi, Suzanne M. Cloonan, Stefan W. Ryter

**Affiliations:** ^1^Lovelace Respiratory Research Institute, Albuquerque, NM 87108, USA; ^2^College of Arts and Sciences, Boston College, 140 Commonwealth Avenue, Chestnut Hill, MA 02467, USA; ^3^Pulmonary and Critical Care Medicine Division, Department of Medicine, Brigham and Women's Hospital, Harvard Medical School, 75 Francis Street, Boston, MA 02115, USA

## Abstract

Heme oxygenase (HO), a catabolic enzyme, provides the rate-limiting step in the oxidative breakdown of heme, to generate carbon monoxide (CO), iron, and biliverdin-IX**α**. Induction of the inducible form, HO-1, in tissues is generally regarded as a protective mechanism. Over the last decade, considerable progress has been made in defining the therapeutic potential of HO-1 in a number of preclinical models of lung tissue injury and disease. Likewise, tissue-protective effects of CO, when applied at low concentration, have been observed in many of these models. Recent studies have expanded this concept to include chemical CO-releasing molecules (CORMs). Collectively, salutary effects of the HO-1/CO system have been demonstrated in lung inflammation/acute lung injury, lung and vascular transplantation, sepsis, and pulmonary hypertension models. The beneficial effects of HO-1/CO are conveyed in part through the inhibition or modulation of inflammatory, apoptotic, and proliferative processes. Recent advances, however, suggest that the regulation of autophagy and the preservation of mitochondrial homeostasis may serve as additional candidate mechanisms. Further preclinical and clinical trials are needed to ascertain the therapeutic potential of HO-1/CO in human clinical disease.

## 1. Introduction

**Figure 1:**
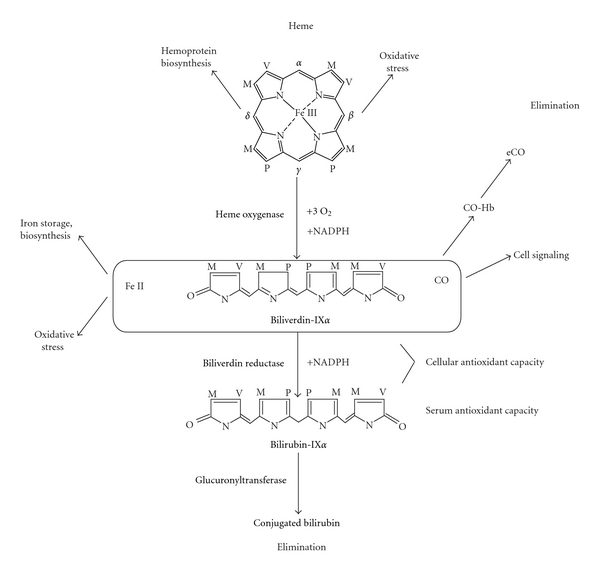
The heme oxygenase reaction. Heme oxygenase-1 catalyzes the rate-limiting step in heme degradation. The reaction produces biliverdin-IX*α*, carbon monoxide (CO), and ferrous iron (Fe II), at the expense of molecular oxygen and NADPH. Biliverdin-IX*α* produced in the HO reaction is then converted to bilirubin-IX*α* by biliverdin reductase. (Side chains are labeled as M: methyl, V: vinyl, P: propionate). The reactants and products of these enzymatic reactions have numerous and diverse biological sequelae. Heme is a vital molecule used in biosynthesis of cytochromes and other hemoproteins. Accumulation of this metabolite may promote deleterious oxidative reactions. Biliverdin-IX*α* and bilirubin-IX*α* may serve as cellular antioxidants, whereas circulating bilirubin may also provide antioxidant benefit in plasma. Bilirubin-IX*α* is conjugated by hepatic glucuronyltransferases and secreted by the biliary fecal route. CO has numerous signal transduction effects as outlined in this review. Systemic CO forms bind hemoglobin to form carboxyhemoglobin (CO-Hb). CO eventually diffuses to the lung where it is eliminated as exhaled CO (eCO). Fe (II) represents a potentially toxic metabolite of heme degradation. A potential metabolic fate of the released iron is sequestration by the iron storage protein ferritin.

Stress-inducible protein systems represent a common and ubiquitous strategy that eukaryotic cells and tissues employ to maintain cellular homeostasis in adverse environments. Of these, the heat shock proteins (HSPs), whose synthesis increases with heat stress, and whose accumulation in turn confers survival advantage to cells undergoing heat stress, were among the first to be identified [1–3]. HSPs act as protein chaperones which play multifunctional roles in protein trafficking and in the clearance of denatured protein aggregates [3]. Although not strictly heat inducible in all cell types, the increased expression of a low-molecular-weight stress protein (32–34 kDa) has emerged as a general response to chemical and physical stress in cultured cells [4–6]. Although the agents that induce this response belong to seemingly disparate chemical and physical classes, a common feature is their potential to evoke cellular oxidative stress (i.e., altered redox homeostasis), and/or to stimulate the inflammatory response [4–10]. The 32–34 kDa protein was identified as identical to heme oxygenase-1 [4], (HO, E.C. 1.14.99.3), a catabolic enzyme, which provides the rate-limiting step in the oxidative breakdown of heme. In the presence of O_2_ and the electron donor, NADPH: cytochrome p-450 reductase, HO converts heme to biliverdin-IX*α*, which is then converted to bilirubin-IX*α* by biliverdin reductase [11] (Figure 1). Additionally, ferrous iron and carbon monoxide (CO) are released during heme degradation [11].

The lung represents a critical organ for toxicological studies, since it provides essential life-sustaining functions in the transfer of molecular oxygen (O_2_) to the circulatory system for ultimate use in respiration and energy generation, and at the same time can act as a major portal of entry for xenobiotic and pathogen exposure [12]. The expression of HO-1 is now believed to act as a general protective mechanism of the lung in response to stress stimuli, especially those involving oxidative or inflammatory components [13–16].

HO-1 has in recent years been demonstrated to confer protection in a number of preclinical animal models of tissue injury and disease [13–20] (reviewed in [21]). This review will highlight those aspects of HO-1 tissue protection relevant to lung disease. Furthermore, accumulating studies over the past decade have shown that the exogenous application of the HO-1 end-product CO, when administered at low concentrations, or alternatively, by pharmacological application of carbon-releasing molecules (CORMs), can also confer protective effects in models of inflammatory stress or tissue injury [22–24] (reviewed in [21, 25]). Tissue protection has also been described for the exogenous application of bile pigments, biliverdin-IX*α*, and bilirubin-IX*α*, which represent the end products of the heme degradation pathway [26–28]. 

**Figure 2:**
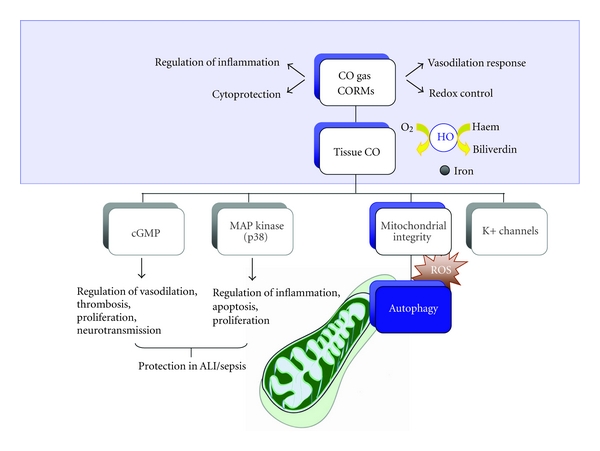
Overview of the signaling pathways relevant to the cytoprotective effects of CO. HO-1 and CO can confer cyto-/tissue-protection in models of acute lung injury (ALI) and sepsis. The homeostatic and beneficial effects of CO gas and CO-releasing molecules (CORMs) in animal models of ALI/sepsis occur through multiple cellular and molecular mechanisms that include regulation of the redox state, inflammation, the vasodilation response. CO gas and CORMs regulate different signaling pathways including cyclic guanosine monophosphate (cGMP), mitogen-activated protein (MAP), kinase signaling pathways, and potassium (K^+^) ion channels. Autophagy is regulated by HO-1/CO levels in a cell-type-specific manner and has a role in the maintenance of mitochondrial integrity and modulation of reaction oxygen species (ROS) production.

Many of the studies concerning HO-1/CO-dependent cytoprotection cite mechanisms involve the modulation of the inflammatory response, including, but not limited to, downregulation of proinflammatory cytokine(s) production [22, 23], as well as the modulation of programmed cell death (i.e., apoptosis)[29, 30], and cell proliferation [31–34], depending on cell type and experimental context (Figure 2). More recent studies, as outlined in this review, suggest additional novel candidate mechanisms for CO-dependent protection, including the regulation of cellular macroautophagy, the maintenance of mitochondrial integrity, and mitochondrial biogenesis. This review will summarize recent findings on the role of HO-1/CO in lung injury and pulmonary disease, with an emphasis on disease pathogenesis and potential therapeutic applications.

### 1.1. The Heme Oxygenase Enzyme System

The microsomal enzyme heme oxygenase (HO, E.C. 1:14:99:3) exerts a vital metabolic function in the regulation of cellular and tissue heme homeostasis and consequently affects intracellular and tissue iron distribution [35]. The HO enzyme was originally discovered (*ca.* 1968-1969) as an NADPH-dependent enzymatic activity present in hepatic microsomal membrane preparations that is responsible for heme degradation [11]. HO is distinct from cytochrome p450, the major hepatic microsomal drug- and steroid-metabolizing system [36]. The two systems share some common features, including a requirement for electron mobilization from the reductase component of cytochrome p450 [37–40]. Similar to cytochrome p450, the HO enzyme reaction utilizes an activated oxygen molecule (O_2_) bound to the ferrous iron of a heme coenzyme to catalyze substrate oxidation [38]. In contrast, p450 oxidizes a bound substrate (steroid or xenobiotic compound) [37], whereas HO specifically degrades heme [11, 41, 42]. The association of heme with the HO enzyme is transient, such that the bound heme uniquely serves as both catalytic cofactor, and substrate [11, 41, 42].

HO catalyzes the selective ring opening of heme at the *α*-methane bridge carbon to form the open chain tetrapyrrole biliverdin-IX*α*. The reaction proceeds through three oxidation cycles, requiring three moles of O_2_ per heme oxidized [11, 43]. In each oxidation cycle, electrons from NADPH are utilized to reduce the heme iron to the ferrous form, which is permissive of O_2_ binding, and subsequently, to activate the bound O_2_ [43]. For each molecule of heme oxidized, one mole each of ferrous iron and carbon monoxide (CO) are also released [11]. In catalyzing the breakdown of heme, HO provides the major source of endogenous biological CO production [11]. The HO reaction, which is rate limiting for the pathway, is generally regarded as a detoxification reaction, in that heme, a potentially deleterious prooxidant is processed for subsequent elimination steps. The cytosolic enzyme, NAD(P)H: biliverdin reductase, reduces biliverdin-IX*α* to the hydrophobic pigment bilirubin-IX*α* [44]. Bilirubin IX*α* accumulates in serum, where it circulates in a protein-bound form, and acts as a physiological antioxidant [45, 46]. Circulating bilirubin IX*α* is conjugated to water-soluble glucuronide derivatives by hepatic microsomal phase II enzymes and then subsequently eliminated through the bile and feces [47].

### 1.2. HO Isozymes

HO can exist in two distinct isozymes: the inducible form, heme oxygenase-1 (HO-1), and the constitutively expressed isozyme, heme oxygenase-2 (HO-2) [48]. The inducible isozyme HO-1 is a ubiquitous mammalian shock protein (identified by molecular-cloning strategies as identical to the major 32 kDa mammalian stress inducible protein) [4]. HO-1 is regulated at the transcriptional level by environmental stress agents. The myriad of inducing conditions that elicit this response is not limited to xenobiotic exposure (i.e., heavy metals, sulfhydryl reactive substances, oxidants) but also includes endogenous mediators (i.e., prostaglandins, nitric oxide, cytokines, heme), physical or mechanical stresses (i.e., shear stress, ultraviolet-A radiation), and extremes in O_2_ availability (hyperoxia or hypoxia), as reviewed in [21, 49]. The induction of HO-1 occurs as a general response to oxidative stress [4, 5, 50]. High levels of HO-1 expression occur in the spleen and other tissues responsible in the degradation of senescent red blood cells [11, 51]. With the exception of these tissues, HO-1 expression is generally low in systemic tissues in the absence of stress. Furthermore, the induction of HO-1 is a common response to elevated temperature in rat organs [52].

The constitutively expressed form, HO-2, is expressed abundantly in the nervous and cardiovascular systems [16]. HO-2 catalyzes the identical biochemical reaction as HO-1 but represents a product of a distinct gene and differs from HO-1 in primary structure, molecular weight, and kinetic parameters [53, 54]. HO-2 contains additional noncatalytic heme-binding domains which are not present in HO-1 [55]. The transcriptional regulation of HO-2 is typically refractory to most inducing agents with the exception of glucocorticoids, which stimulate HO-2 transcription in the nervous tissue [56, 57].

### 1.3. Heme Oxygenase-1: A Cytoprotective Molecule

It is now well established in cell culture and animal studies that HO-1 expression provides a general cyto- and tissue-protective effect, which is elicited as a generalized protective response to environmental derangements. From published studies, it is generally concluded that HO-1 can defend against oxidative stress conditions *in vitro *and *in vivo* by modulating apoptotic and inflammatory pathways [13, 18, 22, 58, 59]. However, the molecular processes and mechanisms, in which HO-1 provides cellular and tissue protection, remain only partially understood. The direct removal of heme may serve an antioxidative function, since heme acts as a prooxidant compound on the basis of its iron functional group [60, 61]. Hypothetically, a buildup of heme from the denaturation of cellular hemoproteins, or from the impaired biosynthesis or assembly of hemoproteins, may result in oxidative stress to the cell, through the promotion of iron-dependent free radical reactions (i.e., Fenton reaction). However, the extent to which the “free” heme pool is mobilized during stress remains unknown. Heme is well known as a lipid peroxidation catalyst in model systems [60, 61] and may cause endothelial cell injury [62]. By breaking down heme, HO liberates heme iron, which can itself represent a deleterious catalytic byproduct with excessive overexpression [63]. HO-derived iron has been shown to drive the synthesis of ferritin, which serves as a protective sink for intracellular redox-active iron [64]. In addition to iron, the reaction products of the HO system, namely, biliverdin/bilirubin, and CO may also contribute to cytoprotection. Evidence for this is based largely on exogenous or pharmacological application of CO or biliverdin/bilirubin as described in detail in the sections below, and it remains incompletely clear whether these mechanisms can account entirely for the cytoprotective properties of the natural enzyme. An emerging consensus is that the pleiotropic effects of HO-1 summarized by the collective effects of the generation and distribution of bioactive products and their downstream sequelae collectively contribute to HO-dependent cytoprotection. In this regard, HO-2 likely also serves as a protective agent against oxidative stress by reducing intracellular heme concentrations and by increasing levels of bilirubin and ferritin, both of which are potent antioxidants [56]. However, HO-2 does not typically respond to transcriptional activation via environmental stimuli, although some posttranscriptional modulation of expression has been described [57, 65].

The critical role of HO-1 in systemic homeostasis was illustrated in the only documented case of HO-1 deficiency in a human subject, who presented with extensive endothelial cell damage, anemia, and abnormal tissue iron accumulation [66]. In addition, knockout mice with the *Hmox1* ^−/−^ genotype revealed hepatic and renal iron deposition, anemia and increased vulnerability to oxidative stress [35, 67].

### 1.4. Biliverdin/Bilirubin Mediators of HO-Dependent Cytoprotection

The cytoprotective effects of HO-1 have been postulated to involve the generation of its end products. The open-chain tetrapyrroles biliverdin and bilirubin exert antioxidant properties *in vitro* [45, 46], which have been demonstrated to confer cytoprotective and antiproliferative properties [27, 28, 68, 69] (reviewed in [70, 71]). Increasing evidence suggests that bilirubin plays an important physiological role as an antioxidant in serum [38, 39]. Increases of serum BR have been correlated with vascular protection and resistance to oxidative stress *in vivo* [72]. Hyperbilirubinemic Gunn rats display reduced plasma biomarkers of oxidative stress following exposure to hyperoxia, relative to normal controls, suggesting that hyperbilirubinemia may confer protection against oxidative stress [72]. Recent clinical studies indicate a relationship between circulating bilirubin levels and risk of vascular disease. Serum BR levels were indicated as an independent, inverse risk factor for coronary artery disease and peripheral vascular disease [73, 74]. In a large-scale prospective study of men, subjects in the midrange of serum BR concentration were at the lowest incidence of ischemic heart disease relative to those subjects displaying the lowest or highest fifth of serum BR distribution [75]. In healthy subjects, serum BR levels were inversely correlated with two indicators for atherosclerosis [76]. Patients with Gilbert's syndrome, who have increased levels of circulating unconjugated bilirubin due to reduced glucuronyltransferase activity, displayed reduced incidence of ischemic heart disease when compared to the general population [77]. Serum samples from Gilbert's patients were further shown to have increased antioxidant capacity and resistance to oxidation [78]. It should be noted that bilirubin also may exert toxicological consequences at supraphysiological levels, as implicated in the neurological injury associated with neonatal jaundice [79].

## 2. Protective Effects of HO-1/CO in Lung Injury and Disease

### 2.1. HO-1/CO in Endotoxemia and Sepsis

HO-1, as an inducible cytoprotective molecule, has been implicated as a modulator of the acute inflammatory response, as demonstrated using *in vitro* and *in vivo* models of inflammatory stress [14, 15, 22]. HO-1 gene expression via adenovirus-mediated gene delivery inhibited the bacterial lipopolysaccharide- (LPS-) induced production of pro-inflammatory cytokines, such as tumor necrosis factor-*α* (TNF-*α*), interleukin-1*β* (IL-1*β*), interleukin-6 (IL-6), and macrophage inflammatory protein-1*β* (MIP-1*β*) in cultured macrophages *in vitro*, and increased the anti-inflammatory cytokine interleukin-10 (IL-10) levels during LPS challenge [22].

HO-1 has also exhibited anti-inflammatory effects through *in vivo* models of inflammatory diseases. Additional studies have shown that enhanced gene expression of HO-1 in rat lungs via intratracheal adenoviral-mediated gene transfer limited murine acute lung injury following influenza virus infection [14] and ameliorated LPS-induced lung injury in mice via increased IL-10 production [15, 22]. Furthermore, administration of biliverdin, a direct product of HO degradation, resulted in a significant decrease of proinflammatory cytokines, such as IL-6, upregulation of IL-10 levels, and reduction of lung injury markers in LPS-treated rats. Thus, biliverdin protected against systemic inflammation and lung injury after lethal exposure to LPS. This defense against LPS-induced injury applied to cultured lung endothelial cells as well as macrophages [80]. HO-1 has also displayed anti-inflammatory effects in various models of tissue injury besides the lung, which include enhanced protection during cardiac [81], renal [82], and liver [83] transplantation.

Several recent studies have implicated a protective role for HO-1 during microbial sepsis [84–87]. Using the cecal ligation and puncture (CLP) technique to induce sepsis, HO-1-deficient mice (*Hmox1* ^−/−^) suffered higher mortality rates compared with HO-1 sufficient mice. These mice were also shown to have an increased level of free circulating heme rendering them more susceptible to death from sepsis [85].

Conversely, targeted overexpression of HO-1 to smooth muscle cells and myofibroblasts, and bowel protected against sepsis-induced mortality associated with *Enterococcus faecalis* infection, enhanced bacterial clearance by increasing phagocytosis and the endogenous antimicrobial response [84].

High-mobility group box-1 (HMGB1) protein can mediate various cellular responses, including chemotaxis and accumulation of proinflammatory cytokines. Thus, this molecule may represent a key target in strategies to limit inflammation. With respect to potential mechanisms for HO-1-mediated protection in sepsis, several studies have demonstrated that circulating levels of HMGB1 contribute to LPS-induced mortality in *Hmox1* ^−/−^ mice [86, 87]. Furthermore, the pharmacological administration of HO-1-inducing compounds (i.e., heme) significantly reduced plasma levels of HMGB1 in mice challenged with LPS or CLP, which was also associated with the reduction of serum TNF-*α*, and IL-1*β* levels [86, 87]. Transfection of HO-1 or induction of HO-1-derived CO resulted in a significant reduction in the translocation and release of high-mobility group box 1 (HMGB1) in CLP-induced sepsis *in vivo*. In conclusion, HO-1-derived CO significantly attenuated HMGB1 release during sepsis, and this inhibition is a necessary step of CO in protection against sepsis [87].

*In vitro* experiments showed that pretreatment with HO-1 inducers, or transfection of HO-1, significantly inhibited HMGB1 release, translocation of HMGB1 from nucleus to cytosol, and release of proinflammatory cytokines (i.e., TNF-*α*, IL-1*β*, and IFN-*β*) in RAW264.7 cells stimulated with LPS. These effects were mimicked by CO donor compounds and reversed by CO scavengers [87]. Thus, inhibition of HMGB1 release via HO-1 treatment may represent a potential application for therapeutic intervention against sepsis [87].

Hemin administration was shown to protect mice from lethal endotoxemia and sepsis induced by LPS or CLP, respectively [87]. In this context, heme administration was used as a pharmacological agent to induce HO-1 in healthy animals before applying sepsis. In contrast however, a recent study has suggested that heme-driven tissue damage contributes to the pathogenesis of severe sepsis. The authors demonstrate that the exacerbated mortality of *Hmox1* ^−/−^ mice subjected to low-grade polymicrobial infection induced by CLP correlated with the accumulation of free heme in the plasma. Administration of free heme to wild-type (*Hmox1* ^+/+^) mice subjected to low-grade microbial infection (nonlethal) was sufficient to elicit a lethal form of severe sepsis. The development of lethal forms of severe sepsis after high-grade infection was associated with reduced serum concentrations of the heme-sequestering protein hemopexin (HPX), a protein produced by the body to scavenge free heme, whereas HPX administration after high-grade infection prevented tissue damage and lethality. Further, the lethal outcome of septic shock in patients was associated with reduced levels of serum HPX concentrations, suggesting that targeting free heme by modulation of HPX might be used therapeutically to treat severe sepsis. Therefore, in a clinical setting, monitoring the patients' levels of circulating heme and/or HPX might be used to predict the likelihood of a fatal outcome in each case of severe sepsis [85].

CO also plays a role in the protection against lung inflammation and injury in rodents. In mice, low doses of CO (250 ppm), as well as HO-1 expression, when administered with a sublethal dose of LPS, selectively inhibited the expression of LPS-induced proinflammatory cytokines including TNF*α*, IL-1*β*, and MIP-1*β* [22]. CO dose-dependently increased LPS-inducible IL-10 [22]. Similar effects were observed in cultured macrophages exposed to CO [22]. The p38 mitogen-activated protein kinase (MAPK) pathway was shown to be important for the CO-mediated effect in these cells [22].

The anti-inflammatory protection against LPS-induced organ injury conferred by CO was also observed in association with inhibition of inducible nitric oxide synthase (iNOS) expression and activity in the lung. In contrast, while CO also protected against LPS-induced hepatic injury, an enhancement of iNOS expression and activity by CO was observed in this organ [88]. Studies of primary lung macrophages and hepatocytes *in vitro* revealed a similar effect; CO inhibited LPS-induced cytokine production in lung macrophages while reducing LPS-induced iNOS expression, and protected hepatocytes from apoptosis while augmenting iNOS expression [88]. It remains unclear to which extent these changes in iNOS contribute to the cytoprotection conferred by CO, as it appears that the functional consequences of iNOS regulation by CO differ in an organ-specific fashion.

Anti-inflammatory effects of CO were also recently demonstrated in a swine model of endotoxin challenge. CO reduced the development of disseminated intravascular coagulation and diminished serum levels of the proinflammatory IL-1*β* in response to LPS and induced IL-10 after LPS challenge [89]. Recent studies evaluated the efficacy of inhaled CO in reducing LPS-induced lung inflammation in cynomolgus macaques (a nonhuman primate model). CO exposure (500 ppm, 6 h) following LPS inhalation decreased TNF-*α* release in the bronchioalveolar lavage fluid (BALF) but did not affect IL-6 and IL-8 release, in addition to reducing pulmonary neutrophilia (not observed at lower concentrations of CO). This reduction of pulmonary neutrophilia was as efficacious as pretreatment with a well-characterized inhaled corticosteroid. However, the therapeutic efficacy of CO required relatively high doses that resulted in high carboxyhemoglobin (CO-Hb) levels (>30%). This work highlights the complexity of interspecies variation of dose-response relationships of CO to CO-Hb levels and to the anti-inflammatory functions of CO [90]. This study is the first to examine the therapeutic index and dose-response relationships of CO therapy in nonhuman primates, and this warrants further investigations in humans [90].

### 2.2. HO-1/CO in High Oxygen Stress

O_2_ is required to sustain aerobic life, but paradoxically, due to its biradical nature and reactivity, and consequently its ability to participate in electron transfer reactions, can also be harmful to life [91]. Supraphysiological concentrations of O_2_ (hyperoxia) are routinely used in the clinic to prevent or treat hypoxemia and acute respiratory failure [92]. However, prolonged exposure to hyperoxia can result in tissue damage in many organs, including lungs, and lead to the development of both acute and chronic lung injury [92]. Hyperoxia-induced damage in mice is characterized by an alveolar-capillary barrier dysfunction, impaired gas exchange, and pulmonary edema [13, 93]. Elevated HO-1 protein expression was reported in lungs of mice and in cultured epithelial cells subjected to hyperoxia [93]. The expression of *ho-1* in rat lungs by intratracheal adenoviral-mediated gene transfer, which increased HO-1 expression in the bronchiolar epithelium, protected against the development of pulmonary damage during hyperoxia exposure [13]. Rats infected with *ho-1* prior to hyperoxia displayed reductions in lung injury markers, neutrophil infiltration, and apoptosis, and a marked increase in survival against hyperoxic stress when compared to control-infected rats [13]. *In vitro*, HO-1 overexpression also protected epithelial cells against hyperoxia-induced cytotoxicity [58].

Similarly, low doses of CO have been shown to provide protection against hyperoxic lung injury. The administration of CO (250 ppm) during hyperoxia exposure prolonged the survival of rats and mice subjected to a lethal dose of hyperoxia and dramatically reduced histological indices of lung injury, including airway neutrophil infiltration, fibrin deposition, alveolar proteinosis, pulmonary edema, and apoptosis, relative to animals exposed to hyperoxia alone [23, 94]. In mice, hyperoxia was shown to induce the expression of proinflammatory cytokines (i.e., TNF*α*, IL-1*β*, IL-6) and activate major MAPK pathways in lung tissue. The protection afforded by CO treatment against the lethal effects of hyperoxia correlated with the inhibited release of pro-inflammatory cytokines in BALF. Genetic studies in mice revealed that the anti-inflammatory effect of CO depended on the MKK3/p38*β* MAPK pathway [94]. Corresponding *in vitro* studies of oxidative lung cell injury have also indicated protective effects of low-dose CO application (250 ppm). CO inhibited hyperoxia-induced apoptosis of cultured epithelial cells, which required the activation of the MKK3/p38*β* MAPK pathway [94] as well as the STAT3 pathway [95]. Further mechanistic studies in pulmonary endothelial cells revealed that low-dose CO application inhibited the initiation and propagation of extrinsic apoptotic pathways in mouse lung endothelial cells subjected to hyperoxia [96]. CO inhibited O_2_-induced activation of the death inducing signal complex (DISC) and downstream activation of apoptogenic factors, including caspases (−8, −9, −3) and Bid, thereby affording protection against cell death. CO also diminished membrane-dependent reactive oxygen species (ROS) production during hyperoxia by inhibiting the ERK1/2 MAPK pathway [96].

### 2.3. HO-1/CO in Ventilator-Induced Lung Injury

Mechanical ventilation is commonly used clinically for the maintenance of critically ill patients. However, this therapeutic tool can lead to the development of acute lung injury (ALI)/and acute respiratory distress syndrome (ARDS). Despite reductions in tidal volume currently implemented during mechanical ventilation in the clinic, the complications of ALI/ARDS continue to present a high rate of mortality (~40%) [97, 98]. The lung damage incurred by mechanical ventilation is referred to as ventilator-induced lung injury (VILI) and involves a sterile inflammatory response to cyclic stretching of the tissue [99]. An anti-inflammatory effect of CO was first described in a two-hit model of VILI in which rats were subjected to an injurious high tidal volume ventilator setting combined with intraperitoneal endotoxin injection. This model caused increased expression of HO-1 in the lung. The inclusion of low-concentration CO (250 ppm) in the ventilator circuit reduced the inflammatory cell count in BALF. In the absence of cardiovascular derangements, CO dose-dependently decreased TNF*α* and increased IL-10 content in the BALF [100]. CO application was also found to confer tissue protection in a mouse model of VILI, using moderate tidal volume settings [101, 102]. In the mouse model, mechanical ventilation caused lung injury reflected by increases in protein concentration, and total cell and neutrophil counts in the BALF. CO reduced ventilation-induced cytokine and chemokine production and prevented lung injury during ventilation, as reflected by the inhibition of ventilation-induced increases in BALF protein concentration and cell count, lung neutrophil influx, and pulmonary edema formation [101, 102]. CO also prevented the HO-1 response to mechanical ventilation, indicating a tissue-protective effect that preceded and did not necessarily depend on secondary activation of stress proteins [101]. Inclusion of CO during ventilation increased the expression of the tumor-suppressor protein caveolin-1 in mouse lung epithelium. Mice genetically deficient in caveolin-1 (*Cav-1* ^−/−^) were reported to be more susceptible to VILI than their wild-type counterparts. Furthermore, CO ventilation failed to confer protection against mechanical ventilation-induced lung injury in *cav-1* ^−/−^ mice, indicating a requirement for caveolin-1 in the protective effects of CO [101]. Mechanical ventilation was also shown to increase the expression of the proinflammatory transcriptional regulator early growth response protein-1 (Egr-1) in the lungs of mice, which in turn was inhibited by CO ventilation. The *Egr-1* ^−/−^ mice resisted lung injury during ventilation, relative to their wild-type counterparts, affirming that Egr-1 acts as a proinflammatory mediator in VILI [102].

In lung macrophages, peroxisome proliferator activated receptor-*γ* (PPAR-*γ*), a nuclear regulator, has been demonstrated to act as an anti-inflammatory mediator by counteracting the proinflammatory effects of Egr-1 [103]. CO exposure was found to increase PPAR-*γ* in cultured macrophages. Furthermore, chemical inhibition of PPAR-*γ* *in vivo* reversed the protective effects of CO in this model with respect to Egr-1 regulation and lung injury parameters [102]. These studies in VILI models are supportive of general protective effects of CO in the maintenance of the alveolar-capillary barrier. CO has also been demonstrated to inhibit alveolar fluid clearance [104], and these effects should also be further studied when implementing CO for pulmonary therapies. These studies collectively suggest that mechanical ventilation in the presence of CO may provide protection in animal models of VILI. Further research is needed to better understand the pathogenesis of VILI as well as the protective potential of CO and other so-called therapeutic gases in these models. It remains unclear whether the protective effects of these gases as observed in the mouse would ultimately translate to clinical effectiveness in humans.

### 2.4. HO-1/CO in Pulmonary Ischemia Reperfusion Injury and Lung Transplantation

The therapeutic potential of HO-1/CO in ischemia/reperfusion (I/R) injury models has been described extensively in rodent systems. Lung I/R caused by occlusion of the pulmonary artery was shown to cause lung apoptosis, as evidenced by biochemical markers including caspase activation, expression changes in Bcl_2_ family proteins, cleavage of PARP, and mitochondrial cytochrome-c release [105]. CO conferred tissue protection in rodents subjected to lung I/R injury, as evidenced by reduced markers of apoptosis, which depended on activation of the MKK3/p38*α* MAPK pathway [106]. Mechanistic studies from the same laboratory revealed that CO conferred similar antiapoptotic protection in cultured pulmonary artery endothelial cells against anoxia reoxygenation stress, which was dependent on activation of the MKK3/p38*α* MAPK pathway [106, 107]. Additional proposed pathway mechanisms included the activation of the phosphatidylinositol-3-kinase/Akt pathway and downstream induction of the signal transducer and activator of transcription (STAT)-3 [107].

*In vivo* studies using homozygous *ho-1* knockout mice (*hmox-1* ^−/−^) demonstrated that HO-1 deficiency conferred sensitivity to the lethal effects of lung I/R injury. Application of exogenous CO by inhalation compensated for the HO-1 deficiency in *hmox-1* ^−/−^ mice and improved survival subsequent to pulmonary I/R [108]. The protection provided by CO involved the stimulation of fibrinolysis, by the cGMP-dependent inhibition of plasminogen activator inhibitor-1, a macrophage-derived activator of smooth muscle cell proliferation [108]. CO also inhibited fibrin deposition and improved circulation in ischemic lungs [109]. These protective effects were related to the inhibited expression of the proinflammatory transcription factor Egr-1, and the subsequent downregulation of Egr-1 target genes, which contribute to inflammatory or prothrombotic processes. The downregulation of Egr-1 depended on the enhancement of cGMP signaling by CO treatment, leading to the inhibition of the ERK1/2 MAPK pathway [109].

I/R injury also represents an important causative component of graft rejection after lung transplantation. During orthotopic left lung transplantation in rats, the transplanted lungs were shown to develop severe intra-alveolar hemorrhage and intravascular coagulation. The application of continuous CO exposure (500 ppm) markedly preserved the graft and reduced hemorrhage, fibrosis, and thrombosis after transplantation. Furthermore, CO inhibited lung cell apoptosis and downregulated lung and proinflammatory cytokine and growth factor production which were induced during transplantation [110]. Additional studies revealed that protection against I/R and inflammatory injury was reduced in syngeneic rat orthotopic lung transplantation by inhalation exposure to either the donor or the recipient [111]. Delivery of CO to lung grafts by saturation of the preservation media reduced I/R injury and inflammation in syngeneic rat orthotopic lung transplantation [112].

### 2.5. Protective Role of CO in Vascular Injury

A protective role for CO in vascular injury has been reported. In this study, inhaled CO prevented arteriosclerotic lesions that occur following aorta transplantation in rodent models. Exposure to a low level of CO (250 ppm) for 1 hour before injury was sufficient to suppress intimal hyperplasia arising from balloon injury [32]. The protective effect of CO was associated with inhibition of graft leukocyte infiltration/activation as well as with inhibition of smooth muscle cell proliferation [32]. A more recent study has shown that intravenous injection of CO-saturated saline caused immediate vasodilation and increased blood flow in the hamster skin microcirculation, an effect that lasted up to 90 mins [113]. These changes were related to increased cardiac output and local cGMP levels. This study supports the possible use of CO-saturated solutions as a vasodilator in critical conditions; however, dosage appears to be critical, since higher and lower dosages by a factor of two were ineffective [113].

### 2.6. Carbon Monoxide and Pulmonary Arterial Hypertension (PAH)

Pulmonary arterial hypertension (PAH) is a terminal disease characterized by a progressive increase in pulmonary vascular resistance leading to right ventricular failure. Several studies suggest that HO-1 or CO can exert protective effects in the context of pulmonary hypertension, and reverse hypoxic pulmonary vasoconstriction. The *hmox-1^−/−^* null mice displayed an exaggerated response to chronic hypoxia relative to wild-type mice, as exemplified by marked right heart hypertrophy, which included right ventricular infarcts and the formation of mural thrombi [114]. Chemical induction of HO-1 inhibited the development of PAH in rat lungs in response to chronic hypoxia [17]. Furthermore, transgenic mice with lung-specific overexpression of HO-1 displayed reduced lung inflammation, pulmonary hypertension, and vascular hypertrophy during chronic-hypoxia treatment, relative to wild-type mice [18]. In monocrotaline- (MCT-) induced hypertension, protective effects were observed by treatment with the antiproliferative agent rapamcyin, which were associated with the induction of HO-1 [115]. *In vitro,* the antiproliferative effect of rapamycin on smooth muscle cells also depended in part on HO-1 expression, as it was diminished in smooth muscle cells derived from *ho-1^−/−^* mice [115].

Inhalation of CO has been shown to attenuate the development of hypoxia-induced PAH in rats, by a mechanism possibly involving activation of Ca^2+^ -activated K^+^ channels [116] and NO generation [34]. In hypoxia and monocrotaline-induced PAH in rodents, daily CO exposure (250 ppm, 1 h) reversed established PAH and right ventricular hypertrophy and restored right ventricular and pulmonary arterial pressures. CO treatment restored pulmonary vascular architecture to a near-normal condition [34]. The protective effect of CO was endothelial cell dependent and associated with increased apoptosis and decreased cellular proliferation of vascular smooth muscle cells [34]. The ability of CO to reverse PAH was further shown to require endothelial nitric oxide synthase (eNOS) and NO production, as indicated by the inability of CO to reverse chronic hypoxia-induced PAH in eNOS^−/−^ mice [34]. Biliverdin and bilirubin have also been shown to exert antiproliferative effects on vascular smooth muscle and thus may also have therapeutic potential in PAH and other diseases involving aberrant vascular cell proliferation [27, 28].

## 3. Role of HO-1/CO in the Regulation of Autophagy

In addition to classical mechanisms such as apoptosis and inflammation, several recent intriguing studies suggest that HO-1, and its byproduct CO, can possibly impact the regulation of autophagy, a vital cellular process, which may in part contribute to the cytoprotective mechanism. Macroautophagy (autophagy) is a regulated cellular pathway for the turnover of organelles and proteins by lysosomal-dependent processing. The autophagy mechanism involves double-membrane vesicles, called autophagosomes or autophagic vacuoles, that target and engulf cytosolic material, which may include damaged organelles or denatured proteins. The autophagosomes fuse with lysosomes to form single-membrane autolysosomes. Lysosomal enzymes facilitate a degradation process to regenerate metabolic precursor molecules (i.e., amino acids, fatty acids), which can be used for anabolic pathways and ATP production [117–124]. This process may thereby prolong cellular survival during starvation. During infection, autophagy assists in the immune response by providing a mechanism for the intracellular degradation of invading pathogens, such as bacteria, and may also contribute to adaptive immune mechanisms [123]. At least 30 autophagy-related (*Atg*) genes have been determined, primarily in yeast. The homologues of many of these Atg genes have been shown to participate in the regulation of autophagy [125, 126]. Among these, Beclin 1 (the mammalian homolog of yeast Atg6) represents a major autophagic regulator [126]. Beclin 1 associates with a macromolecular complex that includes the class III phosphatidylinositol-3 kinase (Vps34). The Beclin 1 complex produces phosphatidylinositol-3-phosphate, a second messenger that regulates autophagosomal nucleation [124, 125]. The microtubule-associated protein-1 light chain-3B (LC3B), the mammalian homologue of Atg8 is an important mediator of autophagosome formation, which is found in association with the autophagosomal membrane [127].

Autophagy has been shown to be both protective and injurious in a variety of different models, suggesting that its role in human diseases is complex. Autophagy is generally considered to be protective when it is induced in response to stress, reducing the activation of lethal signal transduction cascades, and maintaining crucial levels of ATP that allow for the generation of proteins and other biosynthetic reactions. Autophagy also facilitates the elimination of potentially toxic protein aggregates, helping to limit the accumulation of ubiquitinylated proteins that otherwise would inhibit proteasome function. Induction of autophagy affects the progression of the cell cycle (and vice versa), suggesting that autophagy can influence cellular sensitivity to cell cycle-dependent toxins [128].

Autophagy is rarely considered a suicidal mechanism as it usually precedes apoptosis or necrosis [128]. Nevertheless, autophagy has been proposed to contribute to Type-II programmed cell death (PCD), a morphologically distinct form of PCD that involves excess levels of cellular autophagy, degradation of irreversibly damaged organelles, and preservation of cytoskeletal elements. Autophagic cell death occurs during development, in a number of homeostatic processes in adulthood that require the elimination of large amounts of cells, and during the neonatal period in order to maintain cellular energy homeostasis and survival [129]. However, there is still no conclusive evidence that a specific mechanism of autophagic cell death exists, as this phenomenon seems to occur only in cells that cannot die by conventional apoptotic mechanisms [130]. Apoptosis can occur at the same time as autophagy in the same cells suggesting a common regulatory mechanism; however, the precise crosstalk between these two processes remains to be elucidated. Several proapoptotic signaling molecules known to induce autophagy include TRAIL [131], TNF [132], FADD, DRP-I (dynamin-related protein-1), and DAPK (death-associated protein kinase) [133]. Ca^2+^ is a major intracellular second messenger involved in mediating both apoptosis and autophagy, where elevated Ca^2+^ induces autophagy which can be inhibited by ER-associated Bcl-2 [134]. The Bcl-2 proteins are also known to be important in both autophagy and apoptosis signaling. Beclin 1 has been shown to interact with Bcl-2 resulting in the inhibition of Beclin 1-mediated autophagy in response to starvation [135, 136]. Further evidence for a cross-talk between apoptosis and autophagy is also supported by a recent study on Atg5. A truncated form of Atg5 (cleaved by calpains 1 and 2) participates in apoptosis regulation and translocates from the cytosol to mitochondria to trigger cytochrome c release and caspase activation [134]. This Atg5 fragment has been shown to bind to Bcl-X_L_, displacing Bcl-X_L_-Bax complexes, to inactivate Bcl-X_L_ antiapoptotic activity, thereby promoting Bax-Bax complex formation, which suggests that Atg5 may be an independent key player in both apoptosis and autophagy. Functional mitochondria are also needed for autophagic induction [137]. Mitochondria have been proposed to act as a platform for controlling the crosstalk between stress responses, autophagy, and programmed cell death, however, the exact mechanisms through which autophagy can intercept lethal signaling remain unknown.

The role of autophagy, whether protective or deleterious, in human diseases, or specifically in chronic lung disease remains obscure. Recently, we demonstrated a pivotal role for autophagy in cigarette smoke-induced apoptosis and emphysema. We have observed increased autophagy in mouse lungs subjected to chronic cigarette smoke exposure, and in pulmonary epithelial cells exposed to cigarette smoke extract (CSE). Knockdown of autophagic proteins inhibited apoptosis in response to cigarette smoke exposure *in vitro*, suggesting that increased autophagy was associated with epithelial cell death. We have also observed increased morphological and biochemical markers of autophagy in human lung specimens from patients with chronic COPD, suggestive of novel therapeutic targets for COPD treatment [138].

**Figure 3:**
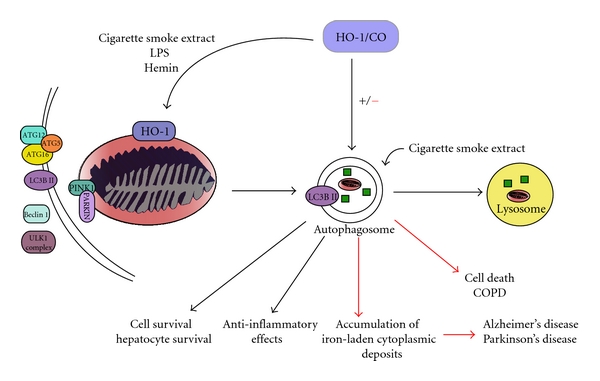
HO-1 as a regulator of autophagy. Autophagic machinery is mobilized in response to stress signals that result in mitochondrial perturbation or accumulations of protein aggregates. A number of proteins have been identified as signaling molecules in preautophagosomal assembly. These include master regulators such as the ULK1 complex, the Beclin-1/Vps34 complex, as well as the autophagic proteins LC3B (Atg8), Atgs 5, 12, 16 which transiently associate with the nascent autophagosome. In inflammation models, HO-1 has been implicated as an inducer of autophagy leading to cell survival and anti-inflammatory effects. In this regard, HO-1 may preserve mitochondrial integrity through the activation of mitochondrial-selective autophagy (mitophagy) which enhances cell survival. In models of neurodegeneration, overexpression of HO-1 leading to activation of autophagy/mitophagy may be detrimental and contribute to neuronal cell death. In lung epithelial cells, HO-1 prevents the induction of autophagy in response to cigarette smoke, leading to cell survival and inhibition of cell death pathways. Overall, the role of HO-1 in controlling cell fate through autophagy is complex. In limited studies to date, the effect of HO-1 on autophagy varies in a cell-type and inducer-specific fashion.

HO-1 has been associated with both the cytoprotective and cytotoxic functions of autophagy induction (Figure 3). HO-1 induces a cytoprotective role for autophagy in lung epithelial cells in response to cigarette smoke by downregulating apoptosis and autophagy-related signaling [139]. CSE increased the processing of LC3B-I to LC3B-II (the lipidated active form), within 1 hr of exposure in Beas-2B cells. Increased LC3B-II was associated with increased autophagic activity, since inhibitors of lysosomal proteases and of autophagosome-lysosome fusion further increased LC3B-II levels during CSE exposure. CSE concurrently induced extrinsic apoptosis in Beas-2B cells involving early activation of death-inducing-signaling-complex (DISC) formation and downstream activation of caspases (−8, −9, −3). HO-1 protected against such CSE-induced effects; adenoviral-mediated expression of HO-1 inhibited DISC formation and caspase-3/9 activation in CSE-treated epithelial cells, diminished the expression of Beclin 1, and partially inhibited the processing of LC3B-I to LC3B-II. These studies were the first to demonstrate a relationship between autophagic and apoptogenic signaling in CSE-induced cell death, and their coordinated downregulation by HO-1 [139].

We have also shown that HO-1 mRNA expression was elevated in the lungs of mice chronically exposed to cigarette smoke [139], implying that HO-1 is upregulated in response to cigarette smoke. In addition, HO-1 was shown to localise to mitochondria in response to hemin, lipopolysaccharide, and CSE in human alveolar (A549), or bronchial epithelial cells (Beas-2B) [140]. These studies suggest that the intracellular location of HO-1, in this case, translocation to the mitochondria may be important for its role in remediating cellular stress and cell death.

In other models, HO-1 has been shown to upregulate autophagy in hepatocytes, leading to protection against hepatocyte cell death and hepatic injury from infection-induced sepsis in mice [141]. HO-1 and autophagy are both upregulated in the liver in response to sepsis and LPS and have been shown to limit cell death. Pharmacological inhibition of HO-1 activity or knockdown of HO-1 prevents the induction of autophagic signaling in this model and resulted in increased hepatocellular injury, apoptosis, and death [141]. Finally, HO-1 dependent autophagic signaling has also been shown to have anti-inflammatory effects in LPS-stimulated macrophages where HO-1 and autophagy collectively serve to limit cytokine production [142]. HO-1 is integral to regulating and dampening the inflammatory response, as demonstrated by the expressed pro-inflammatory phenotype found in HO-1 knockout mice. Many of the anti-inflammatory effects of HO-1 have been attributed to CO which, when provided exogenously, is known to decrease inflammation in macrophages and other cells.

On the contrary, HO-1 has been shown to promote autophagy and consequent cell death in a number of models. HO-1 overexpression results in the activation of mitochondrial-selective autophagy (mitophagy) resulting in the accumulation of iron-laden cytoplasmic inclusions [143] in Alzheimer's disease and Parkinson's disease. HO-1 has also been implicated in the inhibition of autophagosome formation in renal tubular epithelial cells exposed to cisplatin promoting their survival. The absence of HO-1 in renal epithelial cells treated with cisplatin results in impaired autophagy and increased apoptosis. Restoring HO-1 expression in these cells reversed the impaired autophagic response and decreased susceptibility to cisplatin-induced apoptosis, validating the importance of HO-1 expression during cisplatin injury [144]. These data suggest that the role of HO-1 in the control of autophagy is specific to differences in stimulus and cell type; however, in general, HO-1 induction and signaling is an adaptive response to restore cellular homeostasis, much like autophagy. This dual nature of autophagy and HO-1 and the increasing number of pathologies they are associated with highlights of the importance of studying the regulation and effects of autophagy and its control by HO-1 during lung injury.

Our recent studies suggest that CO exposure alone has the potential to induce autophagy in epithelial cells. CO treatment increased the expression and activation of the autophagic protein LC3B in mouse lung, and in cultured human alveolar or bronchial epithelial cells, in a time-dependent manner [145]. Furthermore, CO exposure elicited increased autophagosome formation in epithelial cells, as determined by electron microscopy and GFP-LC3 puncta assays. Recent studies indicate that ROS plays an important role in the activation of autophagy. CO upregulated mitochondria-specific generation of ROS in epithelial cells. Furthermore, CO-dependent induction of LC3B expression was inhibited by the general antioxidant N-acetyl-L-cysteine and the mitochondria-targeting antioxidant Mito-TEMPO, suggesting that CO promotes the autophagic process through mitochondrial ROS generation. We further examined the relationships between autophagic proteins and CO-dependent cytoprotection, using a model of hyperoxic stress. CO protected against hyperoxia-induced cell death and inhibited hyperoxia-associated ROS production. The ability of CO to protect against hyperoxia-induced cell death and caspase-3 activation was compromised in epithelial cells infected with LC3B-siRNA, indicating a role for autophagic proteins [145]. These studies uncover a potentially new candidate mechanism for the protective action of CO in lung cells which has not been previously explored. Further investigations are now underway to investigate elucidate the role of autophagy in lung disease and injury, and in the therapeutic potential of HO-1/CO.

## 4. Pharmacological CO

### 4.1. Carbon-Monoxide-Releasing Molecules

The development of transition metal-based carbon-monoxide-releasing compounds (CORMs) has provided a pharmacological method for delivery of CO as a promising alternative to inhalation. The CORMs used in experimental studies to date include Mn_2_CO_10_ (CORM-1) and the ruthenium-based compounds tricarbonyldichlororuthenium-(II)-dimer (CORM-2) and tricarbonylchoro(glycinato)-ruthenium (II) (CORM-3) [146, 147]. CORM-1 and CORM-2 are soluble in organic solvents, whereas CORM-3 dissolves in water and rapidly releases CO in physiological fluids. A nontransition metallic water-soluble boron-containing CORM (CORM-A1) has also been developed, which slowly releases CO in a pH and temperature-dependent fashion (half-life of 21 min) [148]. This chemical difference dictates how CO causes vasorelaxation and hypotension as CORM-3 elicits a prompt and rapid vasodilatory effect, whereas CORM-A1 promotes mild vasorelaxation and hypotension [149]. Recently, a novel light-sensitive CORM has been developed [150]. Interestingly, and in contrast to inhaled CO, CORMs appear to deliver CO directly to the tissues without significant formation of CO-Hb. There is an abundance of preclinical evidence in large and small animals showing the beneficial effects of CO, administered as a gas or as CORM, in cardiovascular disease, sepsis, and shock; cancer, acute and chronic rejection of a transplanted organ; kidney, liver injury, and some published reports in the acute lung injury field.

The chemistry of transition metals carbonyls is varied, highly versatile, and not restricted to the above-described compounds. Several subclasses of metal carbonyl compounds containing either manganese, iron, cobalt, molybdenum, or ruthenium have been synthesized and tested for their ability to act as CORMs [151]. Though the discovery of these CORM compounds opens up new possibilities, there are still several issues to overcome for medical applications, particularly those in which downstream tissue sites draining the injection site are targeted. These small molecular drugs diffuse rapidly within the body after administration and may liberate CO prior to reaching these target tissues. Thus, there is considerable need for developing a safe and efficient CO-delivery system. Future work in this area should be directed to the synthesis of CORMs which, beyond an effective therapeutic action and low toxicity, need molecular characteristics with appropriate absorption, distribution, metabolism, and excretion properties [25]. A recent report by Kretschmer demonstrates the synthesis of a new CORM (CORM-S1) based on iron and cysteamine, which is soluble in water and releases CO under irradiation with visible light, while it is widely stable in the dark [150]. This is the first example of a light-induced CO release from water-soluble iron-based CORMs, which has low toxicity, compared to that of boron-containing compounds. Hubbell and colleagues have developed micelle forms of metal carbonyl complexes that displayed slowed diffusion in tissues and better ability to target distal tissue drainage sites [152]. The CO release of the micelles was slower than that of CORM-3. CO-releasing micelles efficiently attenuated the LPS-induced NF-*κ*B activation of human monocytes while CORM-3 did not show any beneficial effects. This novel CO-delivery system based on CO-releasing micelles may be useful for therapeutic applications of CO. Efforts in medicinal chemistry development of metal carbonyl compounds are actively ongoing, which should help establish these compounds as a new class of drugs in the near future.

### 4.2. CORMs and Sepsis

CORM compounds are capable of delivering small amounts of CO to biological systems in a controlled manner and are emerging as a potential therapy for sepsis. In terms of lung physiology, most studies to date have focused on the therapeutic effects of CORMs in sepsis models. For example, CORMs reduce cytokine release in LPS-stimulated macrophages [24] and decrease inflammatory response and oxidative stress in LPS-stimulated endothelial cells [153]. *In vivo,* CORMs attenuate systemic inflammation and proadhesive vascular cell properties in septic and thermally injured mice by reducing nuclear factor-*κ*B activation, protein expression of ICAM-1, and tissue granulocyte infiltration [154, 155]. CORM-3 has been shown to prevent reoccurrence of sepsis, CORM-2 prolongs survival and reduces inflammation, while CORM-3 reduces liver injury after CLP [155, 156]. These studies taken together have demonstrated that the CORM-dependent release of CO can reduce mortality in septic mice, suggesting that CORMs could be used therapeutically to prevent organ dysfunction and death in sepsis. As with inhaled CO, full consideration of the toxicological and physiological properties of the released CO, including possible effects on hemodynamics, must be understood before proceeding with CORMs as clinical therapy, with additional considerations for the biological properties of the chemical backbone and transition metal components.

### 4.3. CORM and Ion Channels

Over the last decade, ion channels have been recognized as important effectors in the actions of CO and may play roles in some of the beneficial effects of CO. Members of several ion channel families are molecular targets for the action of CO and/or CORMs and include: (i) the large-conductance, voltage-, and Ca^2+^-activated K^+^ channels [157–163]; (ii) the purinergic P2X2 receptor [164]; (iii) the tandem P domain channel, TREK1 [165]. Interestingly, CORM-2 inhibits the purinergic P2X4 receptor [166] and K_._2.1 [167]. Possible mechanisms by which CO regulates ion channels may include sGC-dependent signaling [168], direct binding of CO to the polypeptide as proposed by Wang and Wu [157], indirect binding via heme [161], or modulation of cellular redox state and mitochondrial function [167, 169]. The precise details of how CO differentially regulates each of these ion channels is beginning to be elucidated but still warrants further investigation and contradictory data has been reported for each channel [170]. For example, the most widely studied ion channel target of CO is the large-conductance, voltage-, and Ca^2+^-activated K^+^ channel, BK_Ca_. While a number of mechanisms have been proposed to explain how CO activates BK_Ca_ channels, the exact mechanism of action is unknown. Direct binding of CO to extracellular histidines has been reported [157] but mutagenesis of these residues did not fully abolish the ability of CO to activate the ion channel [160, 162]. CO has been proposed to bind to a high-affinity, channel-associated heme moiety on the *α*-subunit [160], yet mutation of the key histidine residue required for heme binding does not affect CO activation of the channel [162]. Clearly, further investigation is required to determine the exact mechanisms of action.

Two studies, with opposing outcomes, have reported the regulation of voltage-activated, L-type Ca^2+^ channels. A study by Scragg et al. demonstrated that CO, applied either as the dissolved gas or from the donor molecule CORM-2, inhibits both native (rat) and recombinant (human) cardiac L-type Ca^2+^ channels [169]. This effect arose due to the ability of CO to bind to mitochondria, presumably by interacting at complex IV causing electron leak specifically from complex III. Such leak leads to rapid formation of ROS which causes channel inhibition through a specific interaction with three cytosine residues in the C-terminal tail of the channel's major, pore-forming subunit. Therefore, CO evokes channel modulation in the heart via production of mitochondrial ROS [169]. In another study, the opposite results were reported. Human recombinant intestinal smooth-muscle L-type Ca^2+^ channels were shown to be activated by CO via an NO-dependent mechanism [171]. The reasons for these contrary observations remain unclear but may reflect tissue-specific splice variation of L-type Ca^2+^ channels, as seen for O_2_ regulation of L-type channels [172].

In conclusion, CO modulates ion channels via multiple mechanisms, and it is hoped that these pathways and targets may be exploited for therapeutic intervention in the treatment of a number of important and diverse clinical conditions.

### 4.4. CORM-3 and Mitochondrial Dynamics

The notion that mitochondria serve as important targets in transducing the beneficial signaling properties of CO has been proposed [173]. Recent studies indicate that increased mitochondrial biogenesis is part of the mechanisms by which CO gas and CORMs exert protective effects against cardiomyopathy and cardiac dysfunction in sepsis [174, 175]. Studies by Lancel et al. investigated the potential of CORMs to preserve mitochondrial function in the CLP model of sepsis. CORM-3 treatment in CLP-induced mice prevented the decline in mitochondrial function. Administration of CORM-3 during sepsis also stimulated mitochondrial biogenesis with corresponding increases in (PPAR-*γ*-) coactivator-1*α* protein expression and mitochondrial DNA copy number. CLP was found to impair mitochondrial energetic metabolism and reduce mitochondrial biogenesis in mice [175].

Recent work by Iacono et al. shows that low-micromolar concentrations of CO, delivered to isolated heart mitochondria by the water-soluble CORM-3, uncouple mitochondrial respiration, consequently modulating both ROS production and bioenergetic parameters. In addition, CORM-3 decreased mitochondrial membrane potential at concentrations that did not inhibit cytochrome c oxidase [176]. The CO-mediated effects were attenuated by pharmacological agents known to inhibit mitochondrial uncoupling. Taken together, this work demonstrates that CORM-3, through the liberation of CO, represents a novel regulator of mitochondrial respiration, which in addition to fatty acids and thyroid and steroid hormones could play a crucial role in those pathological conditions for which strategies aimed at targeting mitochondrial uncoupling and metabolism are developed for therapeutic interventions.

## 5. Clinical Aspects of CO

Studies have shown that CO exerts direct anti-inflammatory effects after LPS challenge *in vitro* and in an *in vivo* mouse model [22]. Mice exposed to 250 ppm CO for 1 hour before LPS administration responded with significantly lower levels of proinflammatory cytokines (TNF*α* and IL-1*β*) and higher levels of IL-10 than control mice. As a consequence of this work, the role of CO in various rodent models has since been investigated (reviewed in [25]). On the basis of the rationale provided by these animal studies, Mayr and colleagues studied the effects of CO inhalation on systemic inflammation during experimental human endotoxemia. Specifically, in a randomized, double-blinded, placebo-controlled, two-way crossover trial, experimental endotoxemia was induced in healthy volunteers by injection of 2 ng/kg LPS. The potential anti-inflammatory effects of CO inhalation were investigated by inhalation of 500 ppm CO (leading to an increase in CO-Hb from 1.2% to 7%) versus synthetic air as a placebo for 1 h. CO inhalation had no effect on the inflammatory response as measured by systemic cytokine production (TNF-*α*, IL-6, IL-8, IL-1*α*, and IL-1*β*). In this study, no adverse side effects of CO inhalation were observed [177]. However, given the limited scope of this initial trial, and the protective characteristics of CO application in many animal models of sepsis, further more detailed clinical trials are urgently needed to reach a verdict on the efficacy of CO for reducing inflammation in septic patients. In contrast, a recent clinical trial demonstrates the feasibility of administering inhaled CO to humans with chronic obstructive pulmonary disease (COPD) [178]. In this study, exsmoking patients with stable COPD were subjected to CO inhalation (100–125 ppm for 2 hours/day for 4 days), which increased CO-Hb levels to 4.5%. Inhalation of CO by patients with stable COPD led to trends in reduction of sputum eosinophils and improvement of methacholine responsiveness [178]. In summary, the protective phenotype of CO in rodents in protecting against lung disease has not been recapitulated in human trial studies to date. One possibility is that differences in lung physiological responses to CO exist between different species. Further experiments are required to confirm the safety and efficacy of CO inhalation as a treatment for inflammatory lung diseases.

## 6. Final Remarks

The overexpression of HO-1 by gene transfer has now been shown to confer protection in several models of lung and vascular injury and disease, as well as systemic inflammatory diseases (i.e., sepsis). Potential clinical application of HO-1 would imply targeted gene delivery or pharmacological manipulation of gene expression [179]. The development of vectors for tissue-specific delivery of HO-1 in humans may facilitate gene therapy approaches [179].

 Likewise, similar protective effects have been reported for inhalation CO in models of acute lung injury and sepsis. The demonstrated protective properties of low-dose CO in preclinical rodent models continue to suggest promising therapeutic applications for CO (reviewed in [21, 25, 180]). More recent studies imply the stabilization of mitochondrial function and the stimulation of cellular autophagy as potential candidate mechanisms.

It should be noted that there are limitations, such that some studies have been disputed, and some negative findings reported [181, 182]. However, experimental work showing therapeutic potential of CO has now been extended to large animal models such as swine and nonhuman primates [89, 90].

As an alternative to inhalation of CO, pharmacological application of CO using CORMs may provide a promising therapeutic strategy [25]. Targeted delivery of CORMs may reduce the systemic effects associated with inhaled CO, resulting from CO-Hb elevation, while retaining therapeutic potential. Whether direct application of CO by CORMs administration or inhalation will provide a safe and effective modality for the treatment of human disease requires further research directed at understanding the pharmacokinetics and toxicology of CO or CORMs application in humans [25].

Ultimately, the goal of this experimentation remains to translate the therapeutic potential of CO, whether inhaled or administered through prodrugs, to possible medicinal application in human disease. Although some obstacles remain, limited human experimentation is now underway. Pilot clinical trials to date have indicated either negative efficacy for human CO therapy in endotoxemia or partial efficacy in COPD, while several other trials involving organ transplantation await completion [177, 178]. Currently, new clinical studies in fibrosis and sepsis are projected to begin shortly. Despite the success in animal models, which do not always directly translate to human disease, the therapeutic benefit of CO therapies has yet to be validated in humans.

## Abbreviations

Atg: Autophagy related gene
BALF: Bronchio-alveolar lavage fluid
CLP: Cecal ligation and puncture
CO: Carbon monoxide
CO-Hb: Carboxyhemoglobin
CORM: Carbon monoxide-releasing molecule
COPD: Chronic obstructive pulmonary disease
CSE: Cigarette smoke extract
DISC: Death inducing signaling complex
GFP-LC3: Green fluorescence protein conjugated LC3B
HMGB-1: High-mobility group box 1
HO-1: Heme oxygenase-1
HO-2: Heme oxygenase-2
HPX: Hemopexin
IL: Interleukin
LC3B: Microtubule-associated protein-1 light chain-3B
LPS: Lipopolysaccharide
MAPK: Mitogen activated protein kinase
ROS: Reactive oxygen species
siRNA: Small-interfering ribonucleic acid
VILI: Ventilator-induced lung injury.
